# Correction: National income accounting attributes and economic welfare. Evidence from Pakistan

**DOI:** 10.1371/journal.pone.0320259

**Published:** 2025-03-06

**Authors:** Shuang Yang, Muhammad Waris, Muhammad Kashif Nawaz, Cheng Chen, Ijaz Younis

The first and fourth authors’ names are spelled incorrectly. The correct names are: Shuang Yang and Cheng Chen, respectively. The correct citation is: Yang S, Waris M, Nawaz MK, Chen C, Younis I (2024) National income accounting attributes and economic welfare. Evidence from Pakistan. PLOS ONE 19(8): e0301829. https://doi.org/10.1371/journal.pone.0301829.

The last two authors, Cheng Chen and Ijaz Younis, should be noted as shared co-corresponding authors on this work. Cheng Chen’s email address is: xyzycc@126.com.
